# Advances in Ultrasonic Welding of Thermoplastic Composites: A Review

**DOI:** 10.3390/ma13061284

**Published:** 2020-03-12

**Authors:** Somen K. Bhudolia, Goram Gohel, Kah Fai Leong, Aminul Islam

**Affiliations:** 1 School of Mechanical and Aerospace Engineering, Nanyang Technological University, 50, Nanyang Avenue, Singapore 639798, Singapore; goram001@e.ntu.edu.sg (G.G.); MKFLEONG@ntu.edu.sg (K.F.L.); 2 Institute for Sports Research, Nanyang Technological University, 50, Nanyang Avenue, Singapore 639798, Singapore; 3 Department of Mechanical Engineering, Technical University of Denmark, Produktionstorvet, Building 427A, 2800 Lyngby, Denmark

**Keywords:** thermoplastic composite, ultrasonic welding, energy director, dissimilar materials, bonding strength

## Abstract

The ultrasonic welding (UW) technique is an ultra-fast joining process, and it is used to join thermoplastic composite structures, and provides an excellent bonding strength. It is more cost-efficient as opposed to the conventional adhesive, mechanical and other joining methods. This review paper presents the detailed progress made by the scientific and research community to date in the direction of the UW of thermoplastic composites. The focus of this paper is to review the recent development of the ultrasonic welding technique for thermoplastic composites to thermoplastic composites, and to dissimilar materials. Different ultrasonic welding modes and their processing parameters, namely, weld time, weld pressure, amplitude, type of energy directors (EDs) affecting the welding quality and the advantages and disadvantages of UW over other bonding techniques, are summarized. The current state of the ultrasonic welding of thermoplastic composites and their future perspectives are also deliberated.

## 1. Introduction

Composite materials are considered as the wonder material, as all the industries are obsessed to reduce weight and increase the specific stiffness. Fiber-reinforced composites fit the bill perfectly, and reduce weight significantly. However, there are still some associated obstacles to realize their true potential in the industrial manufacturing landscape. Polymer matrix composites are increasingly used in aerospace, automotive, marine, transport, sports and many other applications, as compared to conventional metals [1,2,3]. This is due to lower weight, specific stiffness, corrosion resistance and high fatigue life, as compared to metals.

Matrix systems used in composites are thermoset and thermoplastic. Recently, thermoplastic composites have become the most demanding material, as these provide numerous advantages over thermoset composites. Thermoplastic (TP) composites are preferred due to their excellent vibration damping [4], high impact resistance [5,6,7,8,9], high productivity, high damage tolerance, fracture toughness [10,11,12], recyclability, reformability, being weldable and repairable, having flexural strength [13,14,15] and their cost-effectiveness compared to thermoset composites [16,17], and these properties attracted its usage for high-end applications, such as manufacturing the fuselage and wing sections of an aircraft. Thermoplastic resin has an inherent ability to become softer once heated above the defined temperature range and retain their properties once they are cooled down. Hence, TP composites are an attractive candidate for the welding of two similar TP composite materials or a TP composite with dissimilar materials, like thermoset (TS) composites and metals. There is a growing call from the wide spectrum of industries (aerospace, automotive, sports and many more) to eradicate the classical ways of joining the polymer composite parts, such as mechanical fastening and the usage of control adhesives. The major drawback of using the former is that composites are susceptible to the high-stress concentration generated due to the holes, and its labor intensiveness whilst the latter require incredibly longer curing time as well as the longer surface preparation [18,19]. Both the conventional approaches of joining hinders the realistic chances of achieving the shorter production cycles, along with lighter weight, and are not suitable for automation processes.

The welding attributes of thermoplastics aids to the cost-effectiveness of the composite part to be manufactured in an industrial environment from forming until the finishing steps [20,21,22,23]. The most feasible welding techniques available for the fusion bonding of thermoplastic composites are resistance [24,25,26], induction [25,27,28,29,30,31,32] and ultrasonic welding [16,23,33,34,35,36,37,38,39,40,41,42,43,44,45,46,47,48,49,50,51,52,53]. They behave differently in a way in which the heat is generated at the welding interface.

Over the last decades, many industries have moved from conventional metals structures to composite structures. As the production costs for manufacturing a finished composite part are high, a technological shift is required in the manufacturing approach. Material selection, the manufacturing process and the finishing steps are the key drivers to reduce the cost of the final composite product. As per the industrial reports [54], the most anticipated research directions in the technological advancement of composite manufacturing technology for automotive, aerospace, sporting, marine, offshore and other applications are:

- Reducing the raw material cost
- Automation of manufacturing for mass production
- Bonding/joining methodologies for complex composite parts
- Recyclability offered by the final composite part
- Repair and structural health monitoring for damage detection

The cost of composite aircraft structures assembled using mechanical fasteners is reported to be 19%–42% of the total aircraft cost [27], thus the effective and optimized fusion bonding will potentially reduce the overall manufacturing costs. A cost comparison study was performed by the Defense and Space Group of the Boeing Company, and it was reported that labor savings of greater than 61% could be obtained by fusion bonding (welding) a composite wing structure, as compared to a bolted one [27]. These traditional methods have many drawbacks [55], such as, in mechanical joints, there is a stress concentration due to holes, there are chances of delamination while drilling the hole, the additional weight of fasteners, rivets and bolts, extensive labor work and the time required [56]. This highlights an intensive and growing need for an improved method of an automated joining of thermoplastic composites. A review study was also carried out by Costa et al., on different fusion bonding technologies that can be implemented in aerospace industries [57]. In the study, the different fusion techniques, such as resistance welding, induction welding, ultrasonic welding, microwave welding, and others, were applied in aerospace structures based on the specific application and requirements.

As explained, there is a growing call from industries to come with a faster and effective way of joining thermoplastic composite structures. In this paper, a detailed literature review is carried out regarding the fusion joining process, ultrasonic welding for composites. The advantages of using ultrasonic welding over conventional and other fusion joining methods and its applications are also deliberated. The important features and the physical parameters deemed important for the ultrasonic welding machine are discussed in detail. The different process parameters that influence the welding quality and other welding aspects for welding TP to TP composites and TP to TS and other materials, are explained in the subsequent sections. A detailed review covering the ultrasonic welding of different TP material systems, and novel attempts to join dissimilar materials, is presented, which could be of significant interest for a scientific and industrial community to refer to. The future directions based on the research gap in the area of ultrasonic welding technology is also discussed in the final section of the paper.

### 1.1. Composite Joining Methods

**Figure 1:**
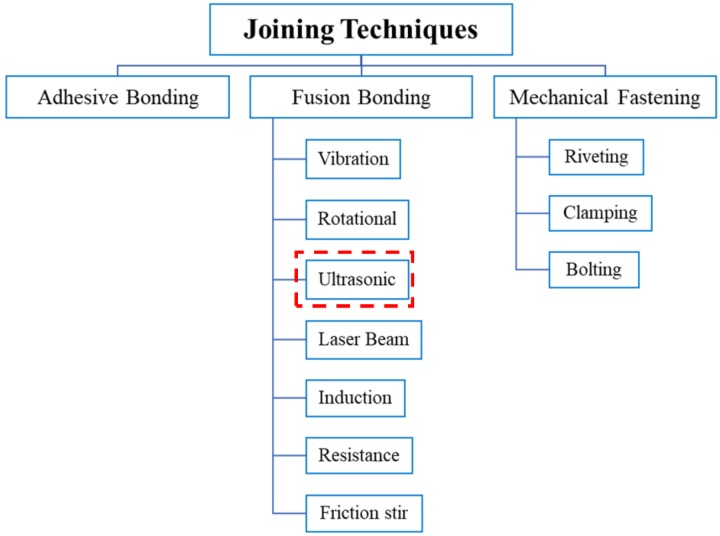
Different joining techniques for composite materials and their corresponding methods.

Manufacturing large composite parts, such as the fuselage and wing section of aircraft, or the body of the automobile car, require a large and complex mold, which consequently means a substantial increase in the cost. However, such a complex part can be manufactured through the assembling of small parts by using different joining techniques. There are several joining methods [58] used for composite materials, as shown in Figure 1. For assembling the large composite structures, the conventional methods which are used in industries are mechanical fastening using rivets and bolts, co-consolidation bonding, and chemical bonding by control adhesives [59].

Mechanical fastening methods, namely bolting, riveting and fasteners, are the most common method of joining in industries. This method is more efficient for metal joining compared to the polymer composites [30]. The benefits of using mechanical fasteners are that no surface preparation required, they are easy to inspect, and can be easily disassembled except in case of rivet joints.

But the major associated drawback is the stress concentration associated due to the holes in the substrates, and it also adds significant weight to the structure. Adhesive bonded joints are one in which two adherends are bonded together by applying the adhesive between them. With the advances in the polymer joining technology, the performance of adhesives in terms of strength, fatigue life and stiffness, have improved. The major advantage of using adhesive joints are that it can be bonded with dissimilar materials, and also has uniform stress distribution and negligible stress concentration. However, adhesive joints also have many drawbacks, such as it requires extensive time for surface preparation and curing, bonding is not as strong as that in the case of mechanical fasteners, and cannot be disassembled [24]. Adhesive joints require a longer curing cycle time, which is not ideal for industrial production. Hybrid joints using both the adhesive and bolted joints are also developed to enhance the mechanical properties. Hybrid joints have shown several advantages in terms of load bearing capability and fatigue life [60,61]. But also, there are several associated disadvantages, such as extensive labor work and longer joining time.

Another option for joining is the fusion bonding, wherein the material at the interface of the joint is heated, diffused and then cooled to get the fused joint. Thermoplastic materials are well known for their weldability [16]. Thermoplastic polymers can be reformed by applying heat and pressure, which makes them suitable for fusion assembly. There are several techniques used for fusion bonding, such as induction, ultrasonic and resistance welding [24,25,62,63]. Resistance welding is a process where joining the interface of the adherend is achieved by resistive heating generated by passing an electric current. It is necessary to place an additional foreign material like a wire or a braid at the interface of the adherend [26,64]. The resistive heat causes the surrounding polymers to melt, followed by the cooling. The main advantage of the technique is its effectiveness to join the large and complex joints. The wire or braid remains the part of the joint, and affects the weld strength. Use of additional foreign material also increases the cost. Induction welding is a joining process wherein an induction coil is moved along the weld line, and the eddy current is induced in the conductive carbon composite laminate, and this results in the melting of the polymer [29,30,31,32]. In the case of laser welding, the partially or fully transparent part has undergone laser treated close to the infrared spectral range [65]. When the light is absorbed by the conductive reinforcement or additives in the adjacent part, the laser energy is transferred into heat, and this finally creates the weld between the two parts. This methodology requires a costly laser system, and is beneficial for mass production approaches. The process though is cleaner, reduces the risk of distortion, is controllable and precise. Vibration or linear friction welding is a process where heat is generated by mechanically moving the parts to be joined under an applied load [66]. Normally, one part is constrained, and there is fast and linear motion of the other part in the plane to the direction of the joint. Rotational welding is a frictional welding which uses the rotational motion and is mostly suitable for the circular joint areas [67].

### 1.2. Advantages and Limitations of Ultrasonic Process 

Many researchers [25,44,67,68,69,70] have studied the applications of ultrasonic welding technology, and explained the advantages and the drawbacks of using this process for thermoplastic composites.

There are many benefits of using the ultrasonic welding technique, as discussed below:

- Ultrasonic welding is one of the fastest joining methods as compared to other techniques, such as induction, resistance welding and arc welding, and hence is most suitable for mass production and automated processes [25].
- No foreign substances such as fillers are required for the ultrasonic welding of specimens [68]. Spot or seam welding can be carried out using this method.
- The surface damage is minimal in ultrasonic welding, as heat is generated at the interface rather than the top of the surface like in other welding processes, such as friction stir welding [69,70].
- It is a clean joining process, as it does not generate fumes or sparks during welding, and thus, is considered environmentally friendly [44].

Some of the limitations of using the ultrasonic process:

- The ultrasonic process is limited to the overlap and shear joints and to the maximum thickness it can weld. As it is difficult for the vibration to penetrate through the thicker parts and the oscillation in the bonding zone, it is not enough to produce a sound quality weld [56]. Currently, the thickness is limited to around 3 mm, due to the power of the equipment being specified [44].
- While using the ultrasonic welding process, the effects of some of the material properties are unavoidable. High stiffness, hardness and the damping factor being the material properties, affect the basis of the ultrasonic technique, which is to convert the vibration into thermal energy. These material properties change the amount of vibration energy required to be delivered to the interface [36].
- Ultrasonic welding works on the principle of mechanical vibration transmission, so audible noise may be produced from the resonance state, and is inevitable. In addition, due to vibrational cyclic loading, the chances of the specimen to fail in fatigue are more [70].

### 1.3. Applications of Ultrasonic Welding

Ultrasonic welding has numerous applications in varied industrial fields. These can be found in automotive, aerospace, medical, electronic and electrical applications and many others [67,71]. In aerospace and automotive industries, they require a lightweight material to increase efficiency by reducing energy consumption. Hence, using a fastener or bolted joint will add the weight, and due to the long curing cycle of adhesive joints, ultrasonic welding is the preferred option for mass production in industries [72]. As mentioned by Palardy et al., ultrasonic welding can be scaled up by sequential welding; i.e., a continuous line of spot welding will serve the same effect of a continuous weld [73]. Thus, ultrasonic welding can be used for applications where a longer part is needed to be welded.

Being a cleaner process as compared to others, and there is no contamination, ultrasonic welding is preferred in medical applications such as hospital wear, medical chip tests, sterile clothing, masks and textiles that are used in clean rooms [74]. Nowadays, this technique is also used in the packaging industries for the packing of items like milk containers; these containers are made from glass, and are sealed with aluminum foil [30]. Furthermore, in electronic industries, it is used to assemble the electronic parts, such as diodes and semiconductors, with substrates. Also, electrical connections between the devices, such as motors, field coils and capacitors, can be connected using ultrasonic welding, for which the traditional fastening and adhesives are not desirable [67,72].

### 1.4. Thermoplastic Polymers for Ultrasonic Welding

There are typically two types of thermoplastic polymer structures: Amorphous and semi-crystalline [75]. High-quality welding of the polymer component is obtained when the polymers are heated to the amount that it reaches its viscous flow state in the contact area [36]. In Amorphous polymers the transmission of ultrasonic vibrations to melt the matrices at the interface are very effective. It has a wide range of load/amplitudes to be welded (Table 1). 

Whereas it is not the same case for semi-crystalline polymers; they are like spring, a percentage of high-frequency vibrations get absorbed internally, and so it becomes hard to transmit the ultrasonic energy to the joint interface. Thus, for the welding of semi-crystalline polymers, a high amplitude vibration is required.

**Figure 2:**
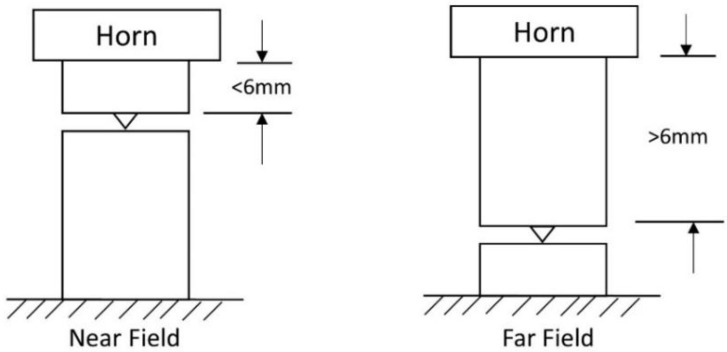
Near and Far-field configurations for the ultrasonic welding of thermoplastic polymers and composites.

Ultrasonic welding of thermoplastics is divided into two categories based on the position of the horn (Figure 2):

- Near-field welding
- Far-field welding

Near field welding refers to a distance of joint interface and the horn to be 0.25 inches (6.35 mm) or less, whereas far-field welding refers to a distance of more than 0.25 inches (6.35 mm) between the joint interface and the horn [45] as shown in Figure 2. Near field welding is suggested for soft and porous thermoplastics, whereas far-field welding is preferably utilized for rigid and amorphous thermoplastics.

In the ultrasonic welding of thermoplastics, heat generation is one of the most important parameters which is generated at the interface resulted from the viscoelasticity phenomenon [33,53]. A protrusion on the polymer surface is required in order to get a good bond as ultrasonic energy is concentrated at the interface. For this, the energy director (ED) of different geometries like semicircular or triangular, are used, as it is a protrusion on one side of the polymer surface. 

Energy directors help to increase the quality of the weld and make the welding process faster and more efficient [67,76,77]. The behavior of polymers was investigated by Lionetto et al., through ultrasonic wave propagation. A glass state of the polymer was defined as the initial state of polymers when they remain stiff, and the temperature is low during the ultrasonic welding. As they are subjected to sinusoidal oscillations, there is an increase in temperature at the interface. The elastic modulus decreases until it reaches glass transition temperature (T_g_) when the temperature is increased, as it requires less force for deformation [78].

There is a change in the form of polymers from the glassy state into a rubbery state in the glass transition region, where the molecular segments become activated. But due to molecular friction, these motions occur with difficulty, and lead to the expansion of the volume of polymers. Increasing the temperature above Tg did lead to a drop in the modulus due to the decrease in viscosity of an amorphous polymer, and at this point, amorphous polymers flow more easily. Whereas semi-crystalline polymers begin to soften above Tg, however, they do not demonstrate fluid behavior until the T_m_ range is achieved [78].

The main polymer characteristics that affect the welding are polymer structure, melt temperature, flowability, stiffness and chemical makeup [75]. Melt temperature is directly proportional to the energy required for welding, higher the melting temperature the more ultrasonic energy is required to weld. Stiffness of the material influences the ultrasonic energy transmission; the stiffer the material, the better is the transmission. Factors like melt temperature and flowability will affect more during the welding of a dissimilar polymer. Due to the difference in melt temperature, the low melting temperature polymer will melt early, and this leads to a poor bond. For better welding of dissimilar polymers, a melting temperature difference should not be more than 22 °C, and it should be chemically compatible with another component [75].

Moisture content also affects the welding quality, as at 100 °C, water will evaporate, and this forms a porous foamy condition and degrades the joint at the interface [79]. Mold release agents are often applied to the surface of the mold cavity to provide an easy de-molding of the parts. But these release agents are transferred to the joint interface, and it interferes with the surface heat generation and obstructs welding [75]. Plasticizers which are added to polymers to impart flexibility can interfere with the ability of the resin to transmit vibration. Impact modifiers, such as rubber, also reduce the weldability of material by lowering the ability of resin to transmit ultrasonic vibrations [53,80,81]. Fillers/extenders constitute a category of additives which enhance the ability of some resins to transmit ultrasonic energy by imparting higher rigidity. However, it is very important to control the amount of filler added. The usage of up to 20% filler has shown positive results in ultrasonic vibration transmission, but adding more may lead to an insufficient amount of resin at the interface, which reduces the welding quality [75].

## 2. Ultrasonic Welding Technique

The ultrasonic welding technology is widely used as a joining technique in industries. It was invented in the 1950s, and is used to join metal, nonmetals, and most recently, the polymer matrix composites [42]. 

### 2.1. Theory of Ultrasonic Welding

Ultrasonic welding falls under the frictional welding category. It is an ultrafast process of joining thermoplastic composites and works on the principle of an application of the high frequency and low amplitude vibration at the interface of the joining surfaces of the adherends to be welded. 

Ultrasonic welding possesses distinct advantages over other fusion bonding methodologies, such that the high weld strength can be obtained, welding can be completed in a few seconds, and it is independent of using a particular material at the interface, as required in resistance and other welding methods. Therefore, ultrasonic welding is getting wide attention in many industrial applications, such as the joining of TP composite parts in the aerospace and automotive industries, wire binding in electronics, and in the packaging industry for sealing purposes.

Ultrasonic welding incorporates the usage of very high-frequency (commonly 20 kHz), and frictional heat is generated at the interface due to the transmission of mechanical vibrations transmitted through thermoplastic adherends. It helps the thermoplastic material to melt and flow and form the interfacial bond between them [30,82]. The main heating mechanisms are viscoelastic friction and surface friction [33,40]. It is deduced from these studies that the interfacial friction is the cause for the initial start of the welding process. Whereas, the viscoelastic heating dominates after the glass transition temperature of the polymer has reached, and it provides the maximum heating during the welding process [40].

**Figure 3:**
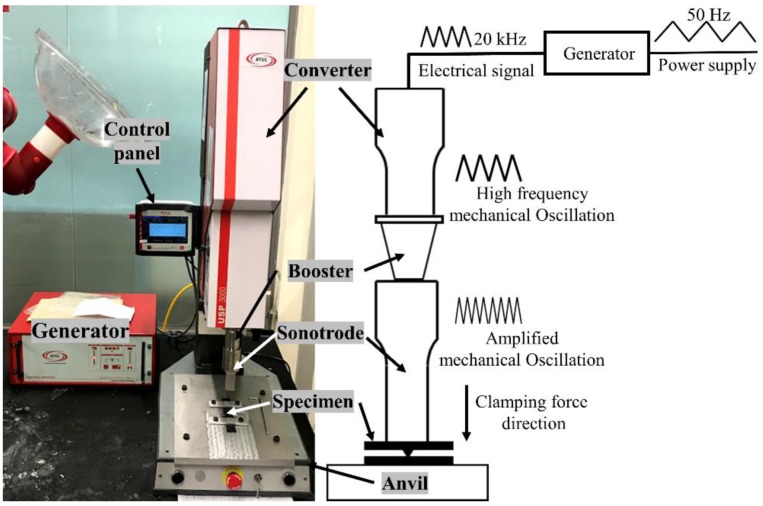
Schematic of the ultrasonic welding process showing the important parts and the associated accessories.

Vibration and solidification phases are two important stages of ultrasonic welding. Ultrasonic heat is generated during the vibration phase upon applying the ultrasonic vibration and mechanical pressure where the matrix system will melt and flow. In the solidification phase, the matrix will consolidate upon only applying the required pressure and time [83]. The specimen is fused as the energy concentration at the interface increases simultaneously with the increase of welding force. Mechanical vibration, welding pressure and solidification pressure are applied to the specimen by the means of a sonotrode. This sonotrode is connected to the transducer, which generates mechanical vibration through the booster. The generated vibration is transmitted to the welding specimen by the required amplification through the booster, and the sonotrode as shown in Figure 3.

In ultrasonic welding, the mechanical oscillation produces an increase in temperature in the bonding zone, and plastics can be reformed with the introduction of heat and pressure. This makes the ultrasonic welding as the best candidate for joining thermoplastic polymers, as the thermoset polymers cannot be reformed with the introduction of temperature or pressure [30,84].

### 2.2. Ultrasonic Welding Equipment

Ultrasonic welding equipment is used to transmit mechanical vibration at high frequency to the joint interface along with a static compressive force. Due to the simultaneous application of static force and dynamic vibrations, fusion at the interface between the specimen occurs, thereby resulting in a weld. There are five main components in the ultrasonic welding machine, such as an ultrasonic generator, a transducer (converter), booster, sonotrode (horn) and the fixture on which specimens rest [35].

#### 2.2.1. Ultrasonic Generator

The main function of an ultrasonic generator is to convert electrical power (5000 W (watts)) at 50–60 Hz into electrical energy at a high frequency of 20 to 40 kHz. The widely used frequency for ultrasonic welding applications is 20 kHz [35].

#### 2.2.2. Transducer

A transducer (converter) functions to convert high-frequency electrical pulses produced from the generator into the mechanical vibrations, and to facilitate this, piezoceramics are used, which expand and contract on exposure to an alternating voltage [35].

#### 2.2.3. Booster

As the name suggests, after obtaining the mechanical vibration from the transducer, a booster is used to increase or decrease the amplitude of vibrations as per the design requirement by adjusting the geometry [35].

#### 2.2.4. Sonotrode

The main component which will directly encounter the specimen is the sonotrode (horn). The function of the sonotrode is to transfer mechanical oscillations to the specimens to be welded. They can be of different designs and geometries to deliver the right/optimum vibrations to the workpiece. This sonotrode is mostly made up of titanium and aluminum and their selection is heavily dependent on the application and possible cost constraints.

#### 2.2.5. Fixture

The fixture is the component placed below the specimen. The main function of the fixture is to hold the specimen in place during the welding process to facilitate a sound bonding. In addition to these five components, an ultrasonic welding machine requires many other components, e.g., mainframe or structure to hold all components together, air compressor, and plc (programmable logic controller) to control the variable parameters.

### 2.3. Types of Ultrasonic Welding

The ultrasonic welding technique is used to join metal to metal, thermoplastics to thermoplastics, and also dissimilar materials like thermoplastics to thermosets, metal-thermoplastic joints [72]. There are basically two types of ultrasonic welding: ultrasonic plastic welding and ultrasonic metal welding. The ultrasonic metal welding process is different from ultrasonic plastic welding technique in two perspectives: (1) how the ultrasonic energy (or vibrations) are transferred to the welding zone, and (2) how the actual weld is created.

**Figure 4:**
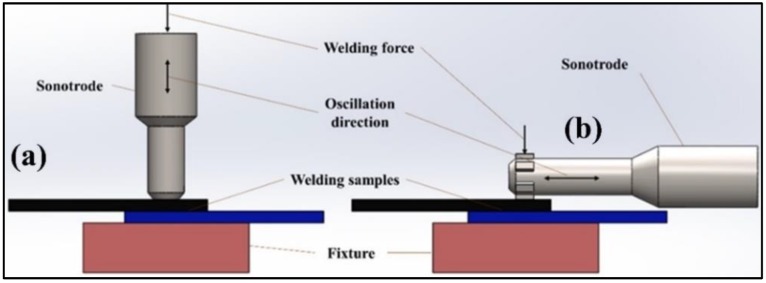
Types of Ultrasonic welding: (**a**) Ultrasonic Plastic welding, (**b**) Ultrasonic metal welding.

In ultrasonic metal welding, vibrations are transmitted to the workpiece in the transverse direction, and pressure is applied on the specimen. This parallel movement enhances the contact area between the horn and the specimen, and the welding is created via the frictional action of the surfaces that creates a solid-state bond without any melting of the material [46,85], as shown in Figure 4. This type of welding technique is preferred for metal joints i.e., metal to metal, and also metals to other materials (pure polymers or polymer matrix composites).

Vibrations delivered at the interface are in the longitudinal direction for ultrasonic plastic welding. For plastic welding, bonding is based on the melting and fusion of the material [30]. During the ultrasonic polymer welding, heat generation at the interface takes place due to the transmission of high-frequency vibrations. It helps the thermoplastic material to melt and flow and form the interfacial bond between them. In ultrasonic polymer welding, oscillation is perpendicular to the welding zone [72]. For welding the similar or dissimilar polymer matrix composites, the principle of the ultrasonic polymer welding technique is used.

**Figure 5:**
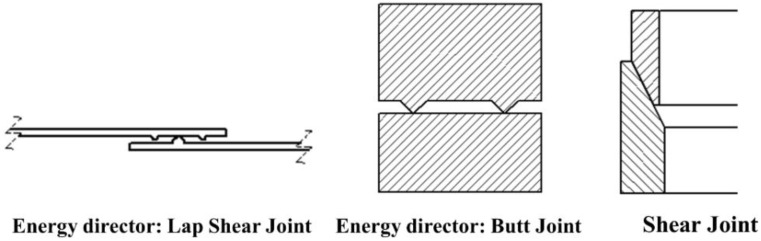
Types of welding joints used in ultrasonic welding technique.

Typically, for ultrasonic welding, there are two types of joints: different types of energy director joints and the shear joints (Figure 5). Energy director (ED) is an extra resin protrusion molded onto the specimen. The joints made using such specimens with ED are called energy director joints. Butt joints and lap joints are the most used energy director joints. Energy director joints are widely used for the plastic welding, i.e., for polymers and polymer matrix composites. Shear joints are designed differently, and herein the direction of vibration transmission is parallel to the welded interface, and the required heat generation takes place due to the frictional shear force at the interfacial region. Shear joint design is generally used for a strong structural or hermetic seal to be obtained and is especially useful for semi-crystalline resins [86,87].

### 2.4. Energy Director

**Figure 6:**
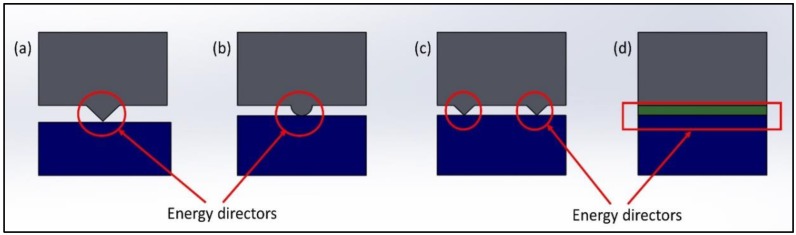
Types of Energy Directors (EDs): (**a**) Triangular ED, (**b**) Semi-hemispherical ED, (**c**) Multiple ED, (**d**) Flat/rectangular ED.

Energy Director (ED) is an important physical parameter in ultrasonic polymer welding. The energy director is a manmade resin protrusion on the composite specimen rich in resin, and does not contain any fibers, and it is placed at the interface during welding, as shown in Figure 6. As ultrasonic welding works on the viscoelastic heating phenomenon at the interface, due to the smaller cross-section of the ED, it helps to increase the viscoelastic heating of the specimen itself by concentrating the energy at the interface [41,88,89]. The design of ED plays a very important role to get a sound welding, since their shape, size, morphology and their configuration, influence the final weld quality. Different shapes of energy directors can be designed, such as semicircular, triangular, rectangular, multiple EDs and others [41,51,90]. They can also be oriented in the parallel or perpendicular direction of welded joints, and their effects could be compared. However, due to the differences in stiffness between the neat polymer and fiber-reinforced composites, it is easy to use the flat energy director. Ultrasonic welding of thermoplastic composites can also be achieved without energy directors with specific process parameters, and by preventing the overheating [91].

Chuah et al. have studied the effect of different types of energy directors on the welding strength of Acrylonitrile Butadiene Styrene (ABS) and Polyethylene (PE) [89]. Fernandez et al. have studied the influence of several configurations of energy directors and investigated the effect of direction of the energy director with respect to the load direction [41]. Goto et al. investigated the shear and tensile strength of the ultrasonic welding of CF/PA6 composite adherends with flat ED (PA6, 0.3 mm film) and without ED at different weld energies [50]. An experimental study based on the Taguchi Method [37,76] was used to study different factors affecting the welding strength, and the studies showed that ED has a greater influence than the welding force and vibration time when joining Nylon 6 composites.

Villegas et al., in one the investigation, has shown that flat ED has no negative impact on the welding process or quality while comparing it with traditional ED (Triangular) [90]. Influence on LSS with and without ED was investigated by Tao et al., using CF/PEEK and ED of 0.45 mm thickness [51]. The result showed that at the same weld time of 0.9 s, LSS showed nearly 50% higher value of adherend welded with ED, as compared to one without ED. According to the study by Liu et al., it was found that the triangular ED is better for virgin polypropylene (PP) and 10% glass fiber-filled PP, but for a higher percentage of fiber-infused composite, semicircular ED was found to have better weld quality [76].

### 2.5. Ultrasonic Welding Parameters

The ultrasonic welding quality is controlled or affected by many welding parameters associated with the welding procedure. Some of the important welding parameters are the vibration amplitude, power, energy, weld time, vertical displacement during welding, applied weld force before, during and after the welding, and the hold time [83,92]. Weld energy, weld time and vertical displacement during welding are mutually exclusive as well. The quality of welding depends upon the amount of input energy [48]. The input energy is calculated by the physical principles of ultrasonic technology, as given by Equation (1) [93]:

(1) E=F×f×A×t

where, E is the Input Energy (J), F the Welding Force (N), f represents Frequency (Hz), A denotes the Vibration Amplitude (μm) and t is the Vibration Time (s).

Harras et al. showed that the optimum joint strength is more related to the total energy input parameter in the welding process than the weld time [34]. Wang et al. showed that the welding energy is one of the dominating parameters to obtain good quality welding by using a two-level full factorial experiment [48]. Short fiber-reinforced composite with Nylon 6 as a resin without ED was investigated by varying the different energy inputs. Results showed that with the increase in welding energy (200J–1000J), there was an increase in bonding efficiency. But with a further increase in welding energy, the bond strength decreases due to the introduction of pores as the horn indentation increases.

Out of these variable parameters, the parameter which significantly affects the welding quality is the amplitude of vibration, weld time and the load applied [51,83,92,94]. The welding load applied, and the vibration amplitude determines the rate at which heat is generated at the interface during welding. The amount of the rate of heat generation is directly proportional to the amount of load and vibration amplitude applied [33].

#### 2.5.1. Weld Time

The ultrasonic welding time is one of the key parameters in the ultrasonic welding process [76]. Tao et al. investigated the effect of different welding times on the welding strength of carbon fiber-reinforced PEEK composite with flat PEEK ED by keeping the other parameters constant [51]. This study showed that the welding quality increases with the increase in weld time from 0.7 s to 0.8 s, but at higher welding time (1.1 s), large cracks and voids are formed at the interface, 0.9 s was found to be the optimum time for good weld quality.

#### 2.5.2. Welding Frequency/Amplitude

Welding frequency also plays an important role in welding quality [91], Tsujino et al. investigated the joining strength at different frequencies of the thermoplastic polymers Polypropylene and Polymethyl methacrylate [38]. This study showed that the joint strength was higher at the high frequency (from 67 to 180 kHz), due to an increase in the vibration velocity, and thereby increasing the temperature at the interface, but joint strength reduces at the lower frequencies (27 and 40 kHz). Ultrasonic generators of 20 kHz frequency are widely used to weld thermoplastic composites, and shown to have excellent welding results [23,39,41,70].

#### 2.5.3. Welding Pressure

The effect of changing the weld parameters (time, pressure, amplitude and type of ED) on the weld quality is also studied by Liu et al., in which they have investigated the weld performance of GF/PP composites [76]. It was reported that the weld pressure has a minimal effect as compared to the weld time, the amplitude of vibrations and type of EDs [76]. 

### 2.6. Welding of TP Composites: Available Test Results

This section presents the available literature on various material systems, such as Thermoplastic composite/Thermoplastic composite, Thermoplastic composite/Thermoset composite and Thermoplastic composite/metal, which are used for ultrasonic welding, and will be explained in detail. Many researches have been carried out on the ultrasonic welding technique by using a different material system for welding, i.e., thermoplastic composites, thermoset composites, metals like aluminum and steel and with different combinations. Selected research advances in ultrasonic welding of the thermoplastic composite are depicted in brief in Table 2 and Table 3.

**Figure 7:**
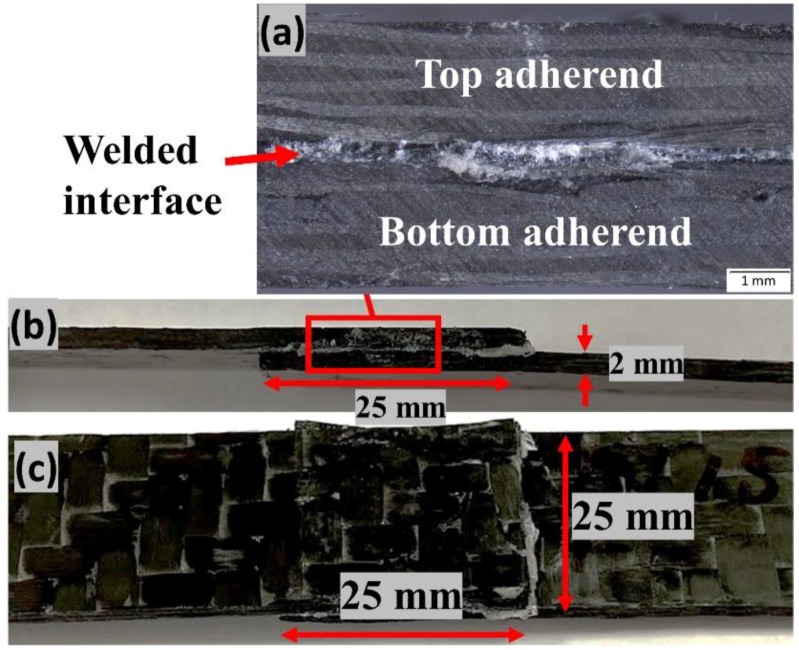
Example of ultrasonically-welded thermoplastic composite (**a**) microscope image of the welded interface (**b**) and (**c**) shows the side and top view of the welded adherends.

Figure 7 shows an example of typical ultrasonically welded thermoplastic composites in the lap shear configuration. Figure 7a also depicts the microscopic image of the welded interface showing the melted and welded matrix layer between the adherends.

#### 2.6.1. Thermoplastic to Thermoplastic (TP-TP) Composites

One of the key benefits of using thermoplastic composite is its weldability property. Many researchers have carried out extensive studies on the welding attributes of different types of thermoplastic composites [62]. Liu et al. [37,76] have investigated the effect of different welding parameters, like weld time, weld pressure, the geometry of the ED, amplitude, hold time and hold pressure on the weld quality using the Taguchi method. The materials used for the study were polypropylene-reinforced glass fiber composites and Nylon 6 reinforced glass fiber composites. Both the investigation showed that weld time, the amplitude of vibration, and the ED geometry has a significant effect on the weld quality.

The energy director has a significant effect on the weld quality, as it allows energy concentration during the joining process [41,88,89]. Chuah et al. [89] investigated the effect of ED by ultrasonically welding pure ABS and PE thermoplastic with different energy director configurations, such as semicircular, triangular and rectangular. The semicircular geometry was found to be the most efficient welding condition, while triangular ED has shown the lowest result. A similar study was carried out by Villegas [41] on the effect of the weld quality by using different configurations, direction and shape of energy director. Polyetherimide (PEI) matrix-reinforced carbon fiber composite was used for the investigation, and the results were examined by carrying out a static lap shear test. The results were examined by lap shear testing, and no significant effect of the direction of the ED on LSS value was noticed. Whereas the laminate with multiple ED showed an increase in welding strength with the increase in the ED volume, up to a certain threshold limit, on the contrary, a further increase in the ED volume resulted in a reduction of 34% in the LSS value comparatively.

**Figure 8:**
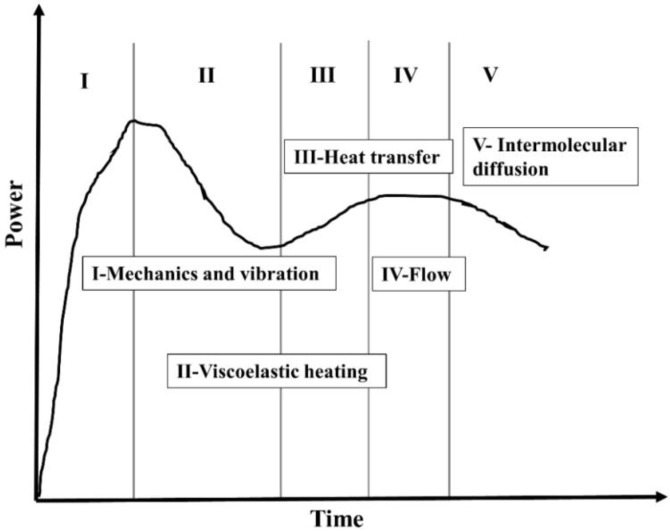
Power curve representing the five stages of ultrasonic welding [83]

A study was carried out by Villegas et al. to use an alternative energy director, i.e., a flat energy director, to investigate its effect on weld quality [83]. In this study, CF/PEI composite was used with flat ED (PEI) of 0.25 mm thickness. The investigation was carried out at different amplitude values and different welding forces. Displacement control mode was used for welding. This research showcased that the vibration phase in the welding process can be divided into five different stages, and showed the relationship of the weld strength w.r.t. all of the vibration phases. Figure 8 represents the five stages in the vibration phase of ultrasonic welding, and the Stage 1 heating of the ED shows the continuous increase in dissipated power until it reaches the maximum value. Stage 2 shows the local melting of the resin, and hence there is a reduction in the power, but the sonotrode displacement remains constant, whereas in Stage 3 there is a sonotrode movement, and it flushes out the molten resin from the interface. Stage 4 shows the plateau in the power curve signaling the local melting of the matrix in the adherend along with the flow of molten ED. In Stage 5, the melting of a matrix in adherend is predominant, which results in a reduction of the power. This possibility was also studied by Benatar and Gutowski on the samples with triangular energy directors, and they analyzed that the changes in the dissipated power and the acceleration are due to the five subprocessES which occur during the ultrasonic welding [108].

A comparison of flat energy director (PPS) and triangular energy director was also studied by Villegas et al., where CF/PPS composite was used [97]. In this investigation, they showed that the ED configuration with triangular strips has two more additional stages as compared to the conventional five stages in the flat ED study. They also showed that the last four stages behave similarly to the flat ED in the power displacement curve.

Similar studies were carried out by Senders et al. [95] and Palardy et al. [73,96] on the effect of flat energy directors, and using the displacement control mode with different material systems. CF/PPS and CF/PEI composites were used for the investigation, respectively. Senders et al. investigated three different welding conditions, with fully welded overlap, spot welding and continuous welding of the adherends [95]. The investigation is to show that zero flow welding can be carried out, i.e., through continuous welding a higher weld strength, which can be obtained without local deformation of the adherends, as the sonotrode moves along the weld line. 

Palardy et al. investigated the effect of the thickness of the flat ED of PEI, 0.06, 0.25 and 0.5 mm on the ultrasonic welding and studied the power curve [96]. This study showed that the higher thickness film i.e., 0.25 and 0.5 mm shows similar behavior as the film melts first and then the substrate. While in the case of 0.06 mm, both the film and adherends melt simultaneously.

Villegas has also investigated the weld time required by the triangular energy director as compared to the flat energy director by comparing its weld strength and using the displacement control mode for testing [97]. The result showed that the melting of the triangular ED is two times faster than the flat ED, but the time required to fill the full overlap area and to consolidate is relatively the same. A study on the ultrasonic welding of the thermoplastic composite without the energy director was also carried out by Zhi et al., where the aim was to find the optimum displacement of the horn using the displacement control method corresponding to the max shear strength [102]. CF/PA-66 composite laminate with 30% weight-fiber was used for the study, and it showed that the displacement time graph can be divided into four stages. The 3^rd^ stage shows the welding of the specimen, and the optimum displacement can be determined. This optimum value yields in the desired welded area and strength. An energy control mode was used by Goto et al. [50], and a constant time mode was used by the author Tao [51], to investigate the welding using the flat ED. A similar study was also carried out by Gao et al. on CF/ Nylon 66 composite laminate to check the weldability of this material system with a 4 mm thickness panel [103]. Results were investigated by manufacturing lap joint laminate without energy director and by varying the weld time and weld pressure. Results showed that it is feasible to weld 4 mm thick panel, and the optimized condition given max weld strength of 5.2 kN was obtained at the welding time of 2.1 s and a horn pressure of 0.15 MPa.

Tao et al. [51] investigated the effect of different welding times on the welding strength of CF/PEEK composites using a flat ED of 0.45 mm thickness. Results from this study showed that with the gradual increase in weld time, weld strength also increases. On the contrary, after an optimum time, a further increase in weld time resulted in larger cracks and voids, and the weld strength was significantly reduced. The reason for this was explained as an increase in weld time gradually melts the material at the interface properly, but excessive increase in weld time leads to the formation of the voids and crack at the heat-affected zone (HAZ). A similar tendency was analyzed by Choudhury et al., where the effect of the welding parameters, such as weld time, hold time and weld pressure were studied [107]. An investigation was carried out on the ultrasonically welded green composite specimens manufactured using bamboo fibers as a reinforcement and poly(lactic) acid (PLA) as a matrix material. The results showed the maximum tensile failure load at the optimum welding condition at welding time of 3 s, hold time of 9 s, and welding pressure of 3 bar [107]. 

Goto et al. [50] investigated the welding strength of CF/PA-6 composite by two tests of lap shear test and cross tensile test with cross-ply and twill woven laminates, and compared the results of both the laminates with and without energy director at different energies. Their study showed that twill woven laminate has higher LSS1, but a lesser LSS2 value, as compared to cross-ply laminate. It also showed that the LSS1 value is around 77% higher for twill woven laminate, with ED as compared to without ED, while LSS2 showed almost the same result. Zhi et al. studied the effect of the weld energy of carbon fiber/polyamide 66 (CF/PA 66) composite laminates on the welding strength. Results from the study showed that an increase in weld energy leads to an increase in weld strength, however, with excessive energy, the weld strength decreases. The main reason for the effect is the thermal decomposition of the composite (decomposition index of 12%–30%) for the deterioration of the weld quality [98]. 

Ultrasonic welding of the GF/PA composite laminate in the shear joint was investigated by Kalyan Kumar et al. [104]. Adherends were welded at different weld time and tested in the lap shear configuration. The result showed to have a maximum separation load at a weld time of 0.6 s with a constant weld pressure of 4 bar and hold time of 0.55 s.

**Figure 9:**
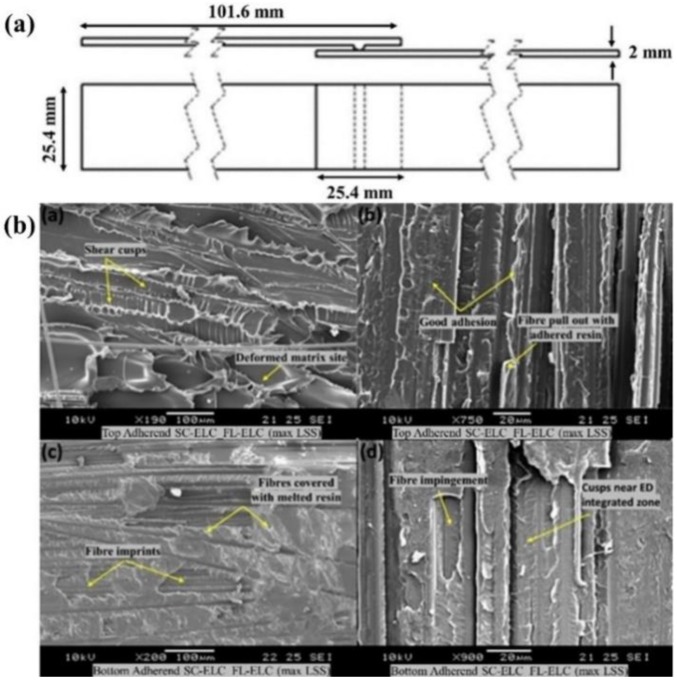
(**a**) Schematic of ultrasonic welding configuration (**b**) Scanning Electron Microscope (SEM) fractography of integrated ED-welded fractures sample at maximum LSS.

Bhudolia et al. recently investigated the ultrasonic welding attributes of the CF/Elium^®^ composite laminates with the integrated semicircular ED (refer Figure 9a) [106]. An optimized study was carried out by varying the weld time and weld pressure at a constant amplitude. The results were analyzed using standard lap shear testing, and were also compared to the conventional adhesive joints. The results showed 23% higher lap shear strength for ultrasonically-welded composite joints, i.e., 17.5 MPa, when compared to the adhesively-bonded joints which were at 14.2 MPa. SEM analysis of the fractured surface at a maximum LSS value was also studied, showing the failure mechanism and features such as shear cusps, plastic deformation sites, good surface adhesion, and the fiber pullout which attributes to the good bonding between the adherends (refer Figure 9b).

**Figure 10:**
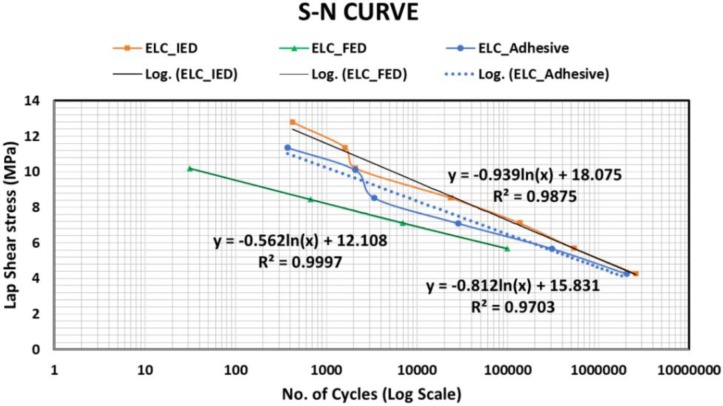
Fatigue test results for adhesive and ultrasonically-welded configurations for CF/Elium^®^ composites [105] with permission from Elsevier.

Recently Bhudolia et al. analyzed the fatigue response of the welded sample and compared the integrated ED, flat ED, and adhesively-bonded sample [105]. The material system studied was carbon fiber-reinforced Elium^®^ composite laminate, and the results showed that the welded samples with integrated ED show a better fatigue life, as compared to adhesives and welded samples with loose film (flat ED) (refer Figure 10). Higher weld strength is attributed to the fiber impingement and shear cusps formation, which contributed to strong interfacial adhesion between the adherend with integrated ED. 

Modeling work is carried out by Wang et al., to predict the Mode II shear by developing a surface-based cohesive model for 40% CF Nylon 6 composite laminates. The model was experimentally verified, and it can be effectively used to predict the composite weld joint properties under shear loading scenarios [48]. Also, detailed research is carried out to understand the influence of moisture absorption on the weld performance of carbon-fiber-reinforced Polyamide 66 composites under a static lap shear test. Even a moisture absorption of 1 wt% was found to be detrimental in reducing the welding property of the composite samples. However, it is also suggested that the reducing effect of weld strength can be fully reversed if the samples are dried before the welding. In addition, there was a minimal effect of moisture absorption when the samples were welded at an amplitude larger than 100 µm [99].

Effect of preheating the CF/polyamide 66 composite samples before welding was studied by Zhi et al., and the tensile and fatigue responses were investigated. While the tensile properties remain unaltered, there was significant improvement noticed for the composite laminates pre-heated at 125 °C with 30% higher endurance limit. Preheating reduced the composite decomposition, and there was a noticeable reduction in the temperature gradient which was attributed to enhanced fatigue performance [100]. Lu et al. also carried out some work on the repair methodology for the welded CF/Polyamide 66 composites. Herein, the welds were repaired by using the second stage of ultrasonic vibration at the same weld parameters, and this resulted in improved quasistatic performance and less scatter in the weld results. The technique yielded positive results owing to the increase in the weld area near the vicinity of the pre-existing weld [101]. Another study is carried out with ABS and HDPE thermoplastics to understand the effect of the height and the angular placement of the energy directors [109]. While the effect of the former was found to be significant, the later did not show much difference in terms of the weld strength of the butt joints and the interfacial temperature [109].

Li et al. also studied the effect of blank holding force (BHF), where an annular clamp was used to apply the variable force [110]. The results showed that the usage of BHF acts as a concentrated energy director, and when used effectively, a localized weld condition is achieved where the melt area is created initially by improvising the point of contact of the sonotrode and the laminate. Recently, Li et al. have also carried out the modeling work, and proposed a model to effectively predict the weld quality based on the correlation between the ultrasonic welding parameters (power and force) and the vibrations [111]. This model can be effectively utilized to predict the online weld quality of carbon fiber composites, and during the automation. 

Recently Palardy et al. also studied the effect of amplitude vibration on ultrasonic welding by carrying out the experimental and modeling work to understand the hammering phenomenon which is caused due to loss of contact between the top adherend and the sonotrode [112]. The proposed model effectively predicts the periodic effect of hammering, which effects the heat generation at the adherend interface. 

Chen et al. carried out research work on the single-sided UW of CF/nylon 6 composites with 30% mass fraction and developed an analytical model to estimate the heat generation during the welding process [113]. This novel welding approach demonstrated higher weld strength and weld area owing to the Coulomb friction at the interface which increased the heat generation during the welding process. The effect of horn misalignment is also studied, where the welding study was carried out on (CF/PA 66) composite. An analytical model was developed to effectively model the horn misalignment effect on the weld size, and the results were experimentally validated [114]. The results showed that misalignment of greater than 4 degrees was an optimal limit after which the weld area and weld strength are dramatically decreased, as there is significant energy deviation at the fraying surfaces.

#### 2.6.2. Thermoplastic Composites to Other Materials

Various researches on the joining of dissimilar material, like the joining of the thermoplastic composite to thermoset composite, thermoset composite to thermoset composite using a thermoplastic coupling layer, and joining of the thermoplastic composite to aluminum and steel, have been investigated. As ultrasonic welding works on the principle of melting of resin and forming the bond between the adherend; the direct welding of thermoset composite is not feasible as they cannot melt or soften owing to their crosslinked molecular structure. Don et al. [115] patented a method of co-curing of the thermoplastic film on the thermoset composite stack, and this method enables to join the thermoplastic composite to the thermoset composite. 

Thermoplastic composite joining to thermoset composite is studied with different thermoplastic films as an interlayer. Some of the investigated polymers are Polyether ether ketone (PEEK) [116], Polysulfone (PSU) [119], Polyphenylene sulfide (PPS), Polystyrene (PS) [120], Polyetherimide (PEI) [118] and Polyvinyl butyral (PVB) [117]. The selection of the coupling film is highly constrained by a multitude of factors, such as the processing temperature, adhesion between the coupling film and the adherend, i.e., compatibility, and other factors [117]. The manufacturing temperature, i.e., the temperature at which the thermoset composite is cured during the co-curing with the film and processing temperature of the coupling film, i.e., the temperature range at which it is welded plays an important role in the bonding and the failure of the adherends. The other two challenges in the welding of the thermoplastic composite to thermoset composite is the bonding between them and the thermal degradation of the thermoset composite because of the high temperature at the interface during welding [116].

Lionetto et al. [117] investigated welding of CF/Epoxy composite laminate to CF/Epoxy laminate using PVB film, which is integrated into the laminate by co-curing it with the CF/Epoxy prepreg. Two film thicknesses of 75 and 250 µm were investigated. Co-curing with the Epoxy laminate was studied, with induction welding and ultrasonic welding, using displacement control mode. The result showed that the LSS value obtained was around 25 Mpa, but the failure was observed in the weld line at the first ply between the PVB film and the reinforced CF. Induction welding gave a thicker weld line, and as a result, the premature failure was observed away from the weld line.

During the fusion bonding of the thermoplastic composite to thermoset composite, there is always a chance of the thermal degradation of the thermoset composite due to the high temperature. Villegas et al. [116] carried out research work to prevent the thermal degradation of the thermoset composite during welding. In the investigation, the major finding was that thermal degradation can be avoided if the welding is carried out under 1 s of time. Additionally, this can be achieved only by the ultrafast ultrasonic fusion bonding process. For the study, the ultrasonic welding of the CF/PEEK and CF/Epoxy material system was used with an ED film of PEEK as an interlayer between the dissimilar adherends. Different configurations were tried for welding, i.e., direct welding without ED, which showed the very poor result, indirect welding, i.e., using PEEK ED film in between the adherends, and also with PEEK coating on the CF/Epoxy laminate. Effective usage of the ultrasonic welding technique with the thin ED film/coating layer provided an efficient heat shield to thermoset laminate, hence thermoset/ thermoplastic laminate can be welded efficiently [121].

Tsiangou et al. [118] investigated the ultrasonic welding of CF/PEI and CF/Epoxy composite by co-curing the coupling layer of thermoplastic PEI with different thicknesses (0.06 and 0.25 mm), and compares it with the loose ED of the same thickness. The results showed that the welding without the loose ED resulted in the overheating of the adherend, showing voids and porosity at the interface. On the contrary, with the loose ED, the adherend gave the good welding strength, and also a good bonding at the interface without any porosity or voids. A similar study for the optimization of welding parameters was carried by the Villegas [49], with CF/PEEK and CF/Epoxy composites with coupling PEI film of 0.05 mm and loose PEI ED film of 0.25 mm thickness. It was concluded that the adherends Carbon/epoxy-PEI and C/PEEK were welded efficiently as the optimal miscibility of PEI and PEEK were exploited during the welding.

As the use of the CFRP in the industries is increasing sharply, and the metals are still widely used in these industries. There is a need for the development of the joining technology of CFRP-metal. Joining methods used conventionally for CFRP-metal are adhesive bonding, bolt connection, riveting and welding [122,123]. Researchers have also investigated the ultrasonic welding of metals to thermoplastics by using a metal welding process, where the applied vibration is parallel to the specimen, as opposed to the plastic welding with the perpendicular vibrations to the surface of the specimen. Balle et al. [39] and Wagner et al. [72] have investigated the ultrasonic welding of Al and Al alloys, respectively, to thermoplastic composites, using both the approaches, and concluded that the metal welding process is an apt solution for joining metals to TP composites as the matrix between the two adherends is removed during the welding process and fibers are directly exposed to the metal surface. Al-Obaidi has investigated the welding of Al with pure ABS polymer and achieved the lap shear strength of 2.3 MPa [124]. There are no other significant studies available in the literature on welding metals to thermoplastic composites.

## 3. Conclusions and Future Directions

With the gradual increase in the usage of the composite materials in aerospace, automotive, sports, wind turbine and other industries, there is a growing call from the industries to develop the novel and fast joining techniques for large and complex structures. In this review article, a detailed literature review was carried out, and it was noticed that fusion bonding has tremendous potential in joining composite parts, and it possesses unique advantages over conventional joining methods. There are several researches carried out on investigating the different fusion bonding techniques, such as ultrasonic welding, resistance welding, friction welding, and others, using different types of thermoplastics composites. These studies showed that the ultrasonic welding technique has more advantages compared to other fusion joining techniques, it is not only an ultra-fast process, but it also provides excellent bonding strength. It is more cost-efficient, as opposed to the conventional adhesive, mechanical and other joining methods. As for larger parts, such as aerospace fuselage, bulkhead, automotive, wind turbine blades, surfboards, etc., adhesive handling is very cumbersome due to the larger curing time and the handling concerns, especially using the manual processes. Researchers have also investigated the welding of different types of thermoplastics, the pure thermoplastic polymers or thermoplastic composites with fiber reinforcements. Different thermoplastics, such as PEEK, PEI, Nylon, PA6, PP, and others have already been investigated. As well as this, welding thermoplastic to thermoset prepreg composites and metals is investigated by many researchers. There are various welding parameters that affect the ultrasonic welding quality, such as weld time, pressure, amplitude, type of Eds, and significant research is carried out in this direction, and is well documented in this review paper. Weld time is generally found to be the dominating parameter which affects the weld quality. From the literature review, it is evident that energy directors also play a very important role on the weld quality.

Although substantial research has been done in the area of the ultrasonic welding of thermoplastic composites, the research is mostly confined to prepreg composites. There is no research carried out on the manufacturing of the integrated EDs using the RTM manufacturing process. Integrated EDs were only manufactured using prepreg composites, and are cured at high temperatures. Also, it will be interesting to further the research to weld the with reactive processing thermoplastics, like Polyurethanes (PU), Cyclic Butylene Terephthalate (CBT), and recently developed acrylic Elium^®^ resin [4,7,14,125,126,127], which can be manufactured using liquid injection processes. Welding of thermoset to thermoplastic composite can be further investigated by exploring more novel manufacturing strategies to create a coating layer on the top of the epoxy laminate to achieve the desired phase change to enhance the lap shear strength. In addition, considerable research should be carried out to understand the weld effectiveness of thermoplastic composites to metals by using different welding strategies, and with different surface preparation techniques for, e.g., the chemical treatment of the metal surfaces to enhance the welding strength. Different demonstrators can be welded ultrasonically for industrial applications, such as bike frames, stringers of the fuselage section, bulkhead structures, and many more, with the integrated ED and with flat ED film approach, and the associated results and challenges can be deliberated from the detailed research. Automation of ultrasonic welding should be investigated with continuous welding of complex structures and comparing its performance with the conventionally used adhesive bonding.

## Figures and Tables

**Table 1:** Structure of amorphous and semi-crystalline thermoplastic polymers.

Amorphous Polymers	Semi-Crystalline Polymers
Distinctive Properties	
Random molecular arrangement	Orderly molecular arrangement
Broad softening temperature/glass transition temperature (T_g_)	Sharp melting point (T_m_)
Easy to thermoform	Difficult to thermoform
Rigid polymers	Soft polymers
Tough, Rigid, Good Creep and Chemical Resistance	Excellent Chemical Resistance
Transparent look	Opaque look
e.g., Acrylonitrile butadiene styrene (ABS), Acrylic, polycarbonate, polystyrene, Polyvinyl chloride (PVC)	e.g., Nylon, Polyethylene terephthalate (PET), Polybutylene terephthalate (PBT), Polyether ether ketone (PEEK), polyethylene

**Table 2:** Summary of selected researches on ultrasonic welding of thermoplastic composites to thermoplastic composites.

Ultrasonic Welding of Thermoplastic Composites to Thermoplastic Composite										
Year	Author	Material System	Factors Studied	ED	Weld Time	Weld Pressure/Force	Amplitude	Frequency	Optimised Weld Strength	Reference
2001	Liu et al.	GF/PP	Effect of ED, weld time and weld pressure	TED, RED, SHED	0.25 s, 0.3 s, 0.33 s	2.5, 3, 3.5 bar	25, 36, 40 µm	20 kHz	13.77 MPa	[76]
2002	Liu et al.	GF/Nylon 6	Effect of ED, weld time and weld pressure	TED, RED, SHED	0.25 s, 0.32 s, 0.4 s	2, 3, 4 bar	25, 36, 40 µm	20 kHz	17.11 MPa	[37]
2010	Villegas et al.	CF/PEI_CF/PEI	NA	Triangular ED	3.5 s	4 MPa	50 µm	20 kHz	32–36 MPa	[41]
2014	Villegas et al.	CF/PEI	Effect of weld pressure	Flat ED (0.25 mm)	Displacement mode	300 N and 1500 N	51.8 and 86.2 µm	20 kHz	37.3 MPa	[83]
2015	Villegas et al.	CF/PPS	Effect of ED	TED, Flat	Displacement mode	1000 N	86.2 µm	20 kHz	37.1 MPa	[90]
2016	Senders et al.	CF/PPS	Effect of ED	Flat ED (0.08, 0.16, 0.24 mm)	Displacement mode	1000 N	86.2 µm	20 kHz	36.5 MPa	[95]
2017	Palardy et al.	CF/PEI	Effect of ED	Flat ED (0.5, 0.25, 0.06 mm)	Displacement mode	1000 N	86.2 µm	20 kHz	37.3 MPa	[96]
2017	Villegas et al.	CF/PPS	NA	TED	Displacement mode	1000 N	86.2 µm	20 kHz	37.1 MPa	[97]
2017	Zhi et al.	CF/PA 66	Effect of weld energy/ thermal decomposition	NA	NA	0.17 MPa	NA	20 kHz	NA	[98]
2017	Zhi et al.	CF/PA66	Effect of moisture content/ Amplitude	NA	NA	0.17 MPa	NA	20 kHz	NA	[99]
2017	Zhi et al.	CF/PA66	Effect of pre-heating	NA	NA	0.17 MPa	NA	20 kHz	NA	[100]
2017	Lu et al.	CF/Nylon 66	Effect of repair on welded joint	NA	NA (Energy controlled mode)	NA	NA	20 kHz	NA	[101]
2018	Zhi et al.	CF/PA 66	Effect of weld time and weld pressure	NA	1.3 s, 1.7 s, 2.1 s, 2.5 s, 2.9 s, 3.3 s	0.13, 0.14, 0.15, 0.17, 0.2 MPa	25 µm	20 kHz	NA	[102]
2018	Gao et al.	CF/Nylon 66	Feasibility to weld 4 mm-thick laminate	No ED	1.3 s, 1.7 s, 2.1 s, 2.5 s, 2.9 s, 3.3 s	0.13, 0.14, 0.15, 0.17, 0.2 MPa	NA	20 kHz	5.2 kN	[103]
2019	Goto et al.	CF/PA6	Effect of energy	Flat ED (0.3 mm)	NA (Energy controlled mode)	940 N	90 µm	15 kHz	40 MPa	[50]
2019	Tao et al.	CF/PEEK	Effect of weld time	Flat ED (0.45 mm)	0.7 s, 0.8 s, 0.9 s, 1 s, 1.1 s	0.3 MPa	25 µm	60 kHz	28 MPa	[51]
2019	Kalyan Kumar et al.	GF/PA	Effect of weld time	NA	0.5 s, 0.55 s, 0.6 s	4 Bar	NA	20 kHz	3.1 kN	[104]
2020	Bhudolia et al.	CF/Elium^®^	Fatigue response	NA	NA	NA	NA	20 kHz	NA	[105]
2020	Bhudolia et al.	CF/Elium^®^	Effect of weld time	Semicircular ED	0.5 s, 1 s, 1.5 s, 2 s	3 bar, 4 bar, 5 bar	48.75 µm	20 kHz	17.5 MPa	[106]
2020	Choudhury et al.	Bamboo fiber/ PLA	Effect of weld time, hold time, weld pressure	NA	1 s, 3 s, 5 s, 7 s, 9 s	1 bar, 2 bar, 3 bar, 4 bar, 5 bar	NA	20 kHz	3.7 kN	[107]

**Table 3:** Summary of selected researches on ultrasonic welding of thermoplastic composites to other materials.

Ultrasonic Welding of Thermoplastic Composites to Other Materials										
Year	Author	Material System	Factors Studied	ED	Weld Time	Weld Pressure/Force	Amplitude	Frequency	Optimised Weld Strength	Reference
2009	Balle et al.	CF/PA66- Aluminum	Effect Al alloys on shear strength	NA	NA/2160 Ws Weld energy	140 N	38–42 µm	20 kHz	30 MPa	[39]
2013	Wagner et al.	CF/PA66- Aluminum	Effect Al alloys and heat treatment on shear strength	NA	NA/2160 Ws Weld energy	Variable	38–42 µm	20 kHz	58 MPa	[72]
2015	Villegas et al.	CF/PEEK-CF/Epoxy	NA	Flat PEEK Film (0.25 mm)	460 ms/830 ms (heating time)	1500 N/300 N	90 µm/ 72 µm	20 kHz	NA	[116]
2018	Villegas et al.	CF/PEEK-CF/Epoxy	Effect of PEI film	Flat PEI Film (0.05 mm (co-cured) + 0.25 mm)	4 s (Solidification)	2000 N	73.4 µm	20 kHz	28.6 MPa	[49]
2018	Lionetto et al.	CF/Epoxy-CF/Epoxy	Effect of co-curing of film of diff. thickness	PVB Film (0.075 and 0.25 mm)	4 s (Solidification)	1500 N	86.2 µm	20 kHz	25 MPa	[117]
2019	Tsiangou et al.	CF/PEI-CF/Epoxy	Effect of co-cured film and lose film	Flat PEI Film (0.06 and 0.25 mm)	4 s (Solidification)	1500 N	86.2 µm	20 kHz	37.7 MPa	[118]

## Abbreviations

UW	Ultrasonic welding
ED	Energy Director
TP	Thermoplastics
TS	Thermosets
ABS	Acrylonitrile butadiene styrene
PVC	Polyvinyl chloride
PET	Polyethylene terephthalate
PBT	Polybutylene terephthalate
PEEK	Polyether ether ketone
PE	Polyethylene
PA	Polyamide
PP	Polypropylene
GF	Glass fiber
CF	Carbon fiber
LSS	Lap shear strength
PSU	Polysulfone
PPS	Polyphenylene sulfide
PS	Polystyrene
PVB	Polyvinyl butyral
PEI	Polyetherimide
HAZ	Heat affected zone
BHF	Blank holding force
CBT	Cyclic Butylene Terephthalate
